# The Relation between Aerobic Fitness, Muscular Fitness, and Obesity in Children from Three Countries at Different Stages of the Physical Activity Transition

**DOI:** 10.1155/2013/134835

**Published:** 2013-02-20

**Authors:** M. Héroux, V. Onywera, M. S. Tremblay, K. B. Adamo, J. Lopez Taylor, E. Jáuregui Ulloa, I. Janssen

**Affiliations:** ^1^School of Kinesiology and Health Studies, Queen's University, 28 Division Street, Kingston, ON, Canada K7L 3N6; ^2^Department of Recreation Management and Exercise Science, Kenyatta University, P.O. Box 43844-00100, Nairobi, Kenya; ^3^Healthy Active Living and Obesity Research Group, Children's Hospital of Eastern Ontario Research Institute, 401 Smyth Road, Ottawa, ON, Canada K1H 8L1; ^4^Institute of Physical Activity, Sport and Health, University of Guadalajara, Avenida Juárez No. 976, Colonia Centro, CP 44100, Guadalajara, JAL, Mexico; ^5^Department of Community Health and Epidemiology, School of Kinesiology and Health Studies, Queen's University, 28 Division Street, Kingston, ON, Canada K7L 3N6

## Abstract

*Background*. The physical activity transition is contributing to an increase in childhood obesity and a decrease in fitness worldwide. This study compared body composition and fitness measures in children from three countries and examined intercountry differences in the relationship between these variables. *Methods*. Participants consisted of 736 Canadian, 193 Mexican, and 179 Kenyan children aged 9–13 years. Body mass index (BMI), waist circumference, triceps skinfolds, aerobic fitness, and muscular fitness were measured. Linear regression was used to examine associations between variables. *Results*. The prevalence of obesity was the highest in Mexican children (9.2% boys, 8.4% girls) and the lowest in Kenyan children (0.9% boys, 2.8% girls). Aerobic fitness (VO_2max_ in mL/kg/min) was the highest in Kenyan children (50.2 boys, 46.7 girls) and the lowest in Canadian children (41.3 boys, 38.3 girls). Aerobic fitness was negatively associated with body composition measures irrespective of country and sex. Mexican children with low aerobic fitness had higher body composition measures than Canadian and Kenyan children. Muscular fitness was not associated with the body composition measures in Kenyan children but was a weak positive correlate of BMI and waist circumference in Canadian and Mexican children. *Conclusion*. The current study provides some evidence to support the physical activity transition hypothesis.

## 1. Introduction

Childhood obesity has reached epidemic proportions [1]. Increases in weight and adiposity at the population level were first observed in high-income Western countries [2]. Research has linked these body composition changes to the nutrition and physical activity transitions which are characterized by an increased consumption of refined and processed foods and decreased levels of physical activity and are closely associated with social and economic changes impacting urbanization, food systems, labour demands, and transportation choices [2–5]. These transitions seem to be occurring simultaneously and low- and middle-income countries are now progressing through them experiencing similar body composition changes to those that have already occurred in high-income countries [6–8]. In fact, in the last decade the prevalence of obesity has tripled in several low- and middle-income countries [9]. As a result, obesity and its related chronic diseases are significant public health issues worldwide [10, 11]. 

In addition to the rise in childhood obesity and inactive lifestyles, secular changes in children's fitness—a strong and independent marker of chronic disease risk [12, 13]—have been documented. Tomkinson and colleagues calculated that the average annual decline in the aerobic fitness of 6–19-year-olds from five geographical regions (Africa, Middle and East Asia, Australia, Europe, and North America) was 0.36% between 1958 and 2003 [14]. There is also evidence from developed countries supporting the notion that childhood obesity and fitness levels are negatively correlated [15]. Whether or not such associations are consistent in developing countries, and whether changes in body composition and fitness at different stages of the nutrition and physical activity transitions reflect those for obesity, requires further investigation. By comparing the body composition and fitness of children living in countries situated at different stages of the nutrition and physical activity transitions, global correlates of childhood obesity can be better understood. By examining the consistency of these correlates across countries, the potential transferability of preventive efforts can be assessed. If correlates are similar from one country to the next, it is likely that comparable factors have contributed to the observed changes and that preventive efforts that work in one country may, if appropriately contextualized, be successful in another. Thus, intercountry comparisons can serve to raise awareness, guide the development of preventive initiatives, and further our understanding of this public health concern. 

The objectives of this study were to (1) compare body composition, aerobic fitness, and muscular fitness measures in children from three countries that currently sit at different stages of the nutrition and physical activity transitions (Canada-end stages, Mexico-mid stages, and Kenya-early stages) and (2) to examine the intercountry differences in the relationships between body composition and fitness measures. 

## 2. Methods

### 2.1. Study Populations

The study population consisted of school-aged children from three countries that currently sit at different stages of the nutrition and physical activity transitions (Canada, Mexico, and Kenya). Canada represents a high-income country that currently sits at the final stage of the transitions as shifts in diet and physical activity occurred decades ago and considerable efforts have been underway for the past decade or so to reverse obesity [2, 3, 6]. Mexico represents a middle-income country that is at the mid-stages of the transitions as changes in dietary intake and physical activity have occurred, but much later than those observed in high-income countries, and only recently has the issue of obesity begun to be addressed [2, 3, 6]. Finally, Kenya represents a low-income country that is at the early stages of the transitions as shifts in diet and physical activity are only beginning to emerge [2, 3, 6].

**Canada**

Canadian participants consisted of a representative sample of 736 children aged 9–13 years who participated in the Canadian Health Measures Survey (CHMS) [16–18]. The CHMS is a nationally representative cross-sectional survey with data collected from 15 sites across Canada between March 2007 and February 2009. Data collection included a combination of a personal interview (demographic information) and a visit to a mobile examination centre for the collection of physical measures, including anthropometry and fitness.

**Mexico**

The study population consisted of a convenience sample of 193 boys and girls from four public schools located in the urban core of Guadalajara, Mexico. Data were collected by our research team at the four schools in November 2009. Children in grades 5 and 6 (10–13 years of age) from the selected schools were invited to participate. Trained personnel directly measured body composition and fitness indicators. An interviewer-administered questionnaire was used to capture demographic details.

**Kenya**

Participants consisted of a convenience sample of 179 school children aged 9–13 from four schools in Kenya. Two of these schools were located in urban areas and two were located in rural areas. Data were collected at the four schools by members of our research team in November 2008. Body composition and fitness data were directly measured by trained personnel. Demographic information was recorded by researchers.

Ethics approval for data collection was granted for all three study populations by respective institutional review boards. Informed consent/assent was also obtained from the child participants and their parents or guardians. 

### 2.2. Data Collection

With the exception of the aerobic fitness measures in Mexico and Kenya, all body composition and fitness data were collected in each country using comparable equipment according to the Canadian Physical Activity, Fitness, and Lifestyle Appraisal (CPAFLA) [19].

#### 2.2.1. Body Composition Measures

Height (to the nearest 0.1 cm) and weight (to the nearest 0.1 kg) were measured by trained personnel using calibrated stadiometers and scales, respectively. These measures were used to calculate body mass index (BMI, kg/m^2^). Subjects were classified into four categories (underweight, normal weight, overweight, and obese) according to the International Obesity Task Force age- and sex-specific BMI cut-points [20, 21]. Triceps skinfolds were measured in duplicate (or triplicate if measures varied by >0.4 mm) to the closest 0.2 mm using Harpenden skinfold calipers (Baty International, UK). Gulick measuring tapes were used to measure the waist circumference, to the nearest 0.1 cm, according to the World Health Organization [22] and CPAFLA [19] protocols (i.e., midpoint between last floating rib and top of iliac crest in the mid-axillary line).

#### 2.2.2. Muscular Fitness

A hand dynamometer (Canada: Takei Scientific Instruments, Japan; Mexico/Kenya: LB9011 Senoh, Japan) was used to measure grip strength in kg. Both hands were measured alternately allowing two trials per hand. The combined maximum score for each hand was calculated. 

#### 2.2.3. Aerobic Fitness

In the Canadian population aerobic fitness was measured using the modified Canadian Aerobic Fitness Test (mCAFT), during which children had to complete one or more 3-minute “stepping” stages (up and down steps with increasing intensity) at predetermined speeds based on their age and sex [19]. Children aged 6–14 years started at what is stage five for women to a maximum of three stages [23]. Participants' heart rate was recorded after each stage, and the test was completed when it reached 85% of their age-predicted maximal heart rate (220 − age). Predicted maximal aerobic power (VO_2max_) was calculated for all participants using the pediatric-specific equation VO_2max_  (mL/kg/min) = 3.23  (OC) − 1.31  (BMI) + 1.39  (age) − 49.21, where OC is the oxygen cost of stepping [24]. Other equations suggested specifically for adults using the mCAFT were not used as these have not been validated on children [19, 25, 26]. 

In the Mexican and Kenyan populations the 20 Metre Shuttle Run Test was used to measure aerobic fitness [27]. This test involved continuous running by participants between two lines 20 metres apart in time to recorded beeps on a compact disc. The participants continued running between the two lines, turning when signalled by the recorded beeps. Each minute, a sound indicated an increase in speed and the beeps became closer together. If children did not reach the line in time for each beep, the child had to run to the line, turn, and try to catch up with the pace within two more beeps. The test was stopped when the child failed to reach the line (within 2 metres) for two consecutive ends. The level at which the child ended the test was recorded, and Leger's equation [28] was then used to calculate peak oxygen consumption (VO_2max_). This test is currently the most widely used aerobic fitness field test within children and adolescents [27] and has been shown to be a reliable and valid method of estimating VO_2max_ in this age group [14].

### 2.3. Statistical Analysis

All analyses were performed using SAS version 9.1 (SAS Institute, Cary, NC, USA). Data were analyzed separately by sex and country of origin. Estimates of means and their associated 95% confidence intervals were produced for all measures. Pearson correlations were completed between the three body composition measures within each sex and country subgroup. Linear regression models were used to examine the associations between the body composition (BMI, triceps skinfold, and waist circumference), aerobic fitness, and muscular fitness variables. Age was included as a covariate in these models. Regression diagnostics showed that residuals of the dependent variables (BMI, triceps skinfold, and waist circumference) were normally distributed, and thus no transformations were needed. Differences in the descriptive and regression analyses across countries were determined by examining whether 95% confidence intervals of the means and regression (intercepts and coefficients) overlapped. Because of the complex sampling strategy, bootstrapping techniques were used on the Canadian data to generate the confidence intervals [29, 30]. 

## 3. Results

### 3.1. Descriptive Statistics

Descriptive statistics are shown in Table 1. The mean age of children in all three countries was 11 years. There were no differences in the mean height of girls in all three countries, but the mean height of Kenyan boys was less than that of Canadian and Mexican children. The mean BMI, waist circumference, and skinfold values of Canadian and Mexican boys and girls were higher than those of their Kenyan counterparts. There were no differences between Canadian and Mexican children for these three body composition measures with the exception of waist circumference, which was higher in Mexican boys. The prevalence of obesity was highest in Mexican children while the prevalence of underweight was highest in the Kenyan children. No differences between countries were observed for grip strength. However, aerobic fitness (VO_2max_) was different in boys across all three countries with the Kenyan's having the highest values and the Canadian's having the lowest values. In girls, aerobic fitness scores were higher in Kenya and Mexico than in Canada. 

### 3.2. Associations between Body Composition Measures

Table 2 shows the correlations between the three body composition measures within each sex and country subgroup. Correlation coefficients were quite strong (*r* value range of 0.62–0.95), irrespective of sex and country. The correlations in Kenyan boys and Canadian girls tended to be weaker than in the other sex and country subgroups. BMI tended to be more strongly correlated with waist circumference than triceps skinfold, regardless of sex and country. 

### 3.3. Associations between Body Composition and Aerobic Fitness Measures

**Figure 1:**
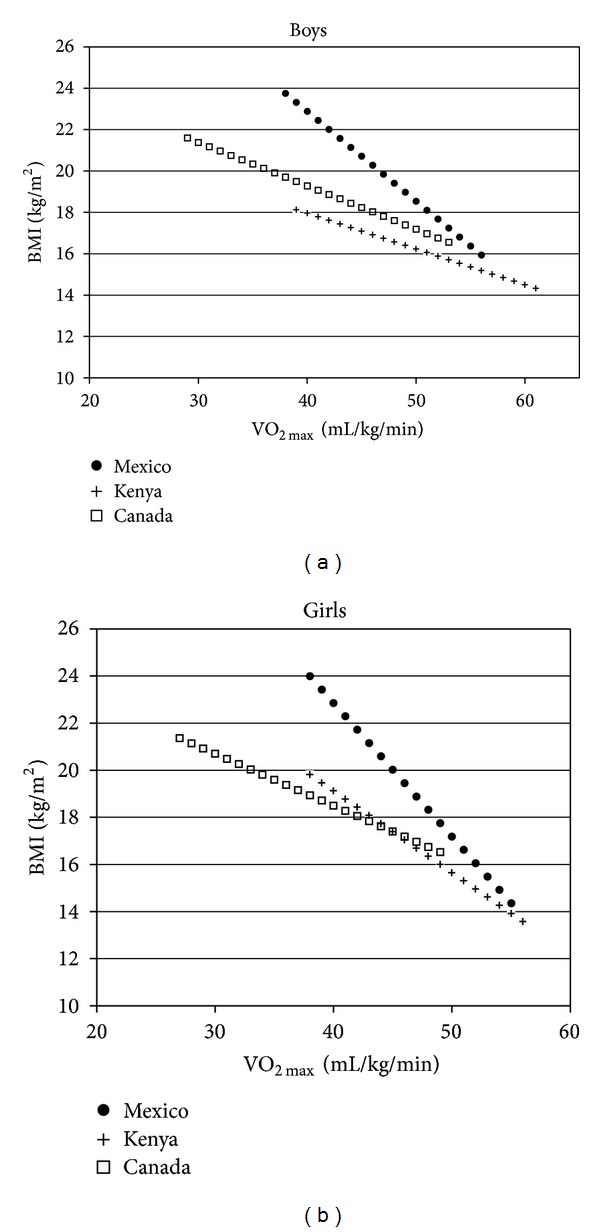
Association between aerobic fitness and body mass index (BMI) in boys (a) and girls (b) from Canada, Mexico, and Kenya. The data for each sex and country subgroup are plotted from 2 SD below the mean to 2 SD above the mean. The figure shows a negative association irrespective of sex and country. The figure also displays that the intercepts and slopes of the regression lines are greater in Mexican children than in Canadian and Kenyan children. Thus, for BMI, Mexican children with low aerobic fitness levels have higher body composition values than do Canadian and Kenyan children. However, body mass index values of children in all three countries are similar in those with high aerobic fitness levels.

Table 3 shows the results from the age-adjusted linear regression analyses looking at the association between aerobic fitness and the three body composition measures. The table displays the slopes (beta-coefficient) of the regression lines, the model fit (*R* ^2^), and the predicted BMI at a VO_2max_ of 40 and 50 mL/kg/min for each sex and country subgroup. The overall patterns of findings indicate the following: (1) aerobic fitness and body composition measures were negatively associated irrespective of country, sex, and body composition measure examined. (2) The slopes of the regression lines and predicted BMI at a low aerobic fitness (e.g., 40 mL/kg/min) tended to be greater in Mexican children than in Canadian and Kenyan children. Thus as illustrated in Figure 1 for BMI, Mexican children with low aerobic fitness levels had higher body composition values than did Canadian and Kenyan children. However, the body composition values of children in all three countries were similar in those with high aerobic fitness (e.g., 50 mL/kg/min). (3) The *R* ^2^ values for both sexes were higher in Canadian children (range 0.37–0.53) than in Mexican children (range 0.31–0.37) and higher in Mexican children than Kenyan children (range 0.11–0.32). Thus, aerobic fitness was more strongly associated with obesity in the most developed country (Canada) and least strongly associated with obesity in the least developed country (Kenya).

### 3.4. Associations between Body Composition and Muscular Fitness Measures

**Figure 2:**
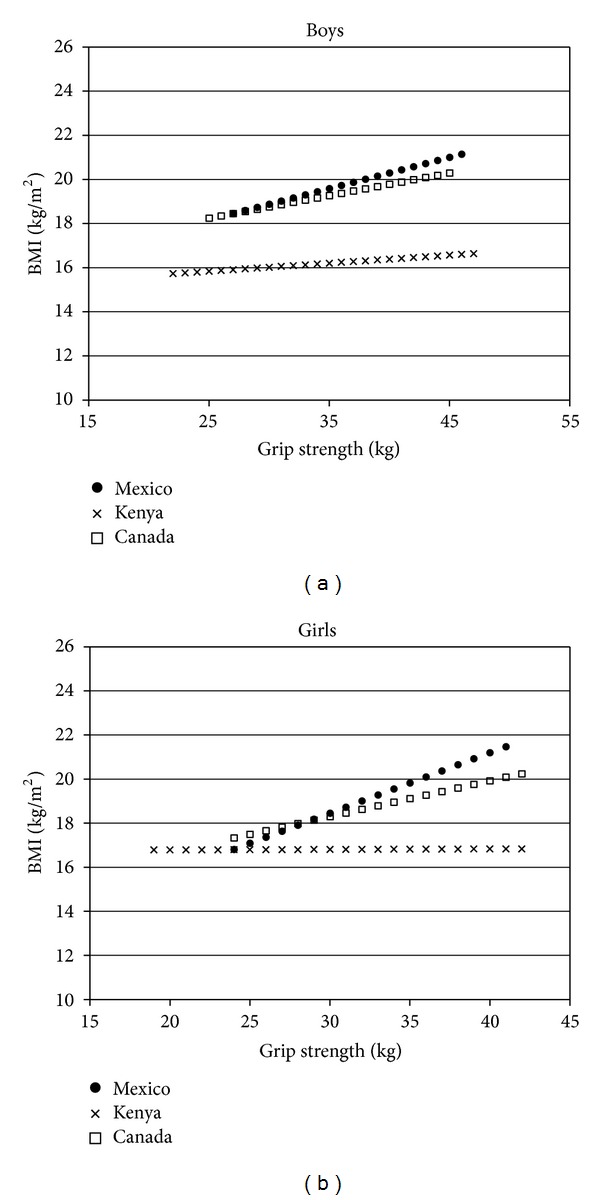
Associations between muscular fitness (grip strength) and body mass index (BMI) in boys (a) and girls (b) from Canada, Mexico, and Kenya. The data for each sex and country subgroup are plotted from 2 SD below the mean to 2 SD above the mean. The figure shows that muscular fitness is positively associated with BMI in boys and girls from Mexico and Canada. The association is less pronounced and not statistically significant in Kenyan boys and girls.

Table 4 and Figure 2 show the results from the linear regression analyses looking at the associations between the muscular fitness (grip strength) and body composition measures. The overall findings indicate the following: (1) muscular fitness was not associated with any of the body composition measures in boys and girls from Kenya. (2) Muscular fitness was positively associated with BMI and waist circumference, but not skinfold thickness, in boys and girls from Canada and Mexico. These associations were weak (*R* ^2^  range = 0.09–0.14) in Canadian children and Mexican boys and were of a modest strength (*R* ^2^ = 0.32) in Mexican girls.

## 4. Discussion

The results provide supporting evidence of intercountry differences in the aerobic fitness and body composition of children from countries at different stages of the nutrition and physical activity transitions. Negative relationships between aerobic fitness and obesity were observed in boys and girls from all three countries; however, these relationships were more pronounced in Mexican children than in Canadian and Kenyan children. 

Differences in aerobic fitness were observed across all three countries wherein Kenyan children were the most fit and Canadian children were the least fit. Although mixed results have been reported in the literature (possibly resulting from the use of invalid self-reported physical activity questionnaires [31]), evidence based on valid questionnaires, objective physical activity measures, and physical activity interventions suggest that aerobic fitness reflects the amount of aerobic physical activity performed in recent weeks and months [32–34]. Thus, results from the current study are consistent with each country's current stage within the physical activity transition. These results are also supported by Tomkinson and Olds who compared the secular decline in the aerobic fitness of 6–19-year-old children from 27 countries in recent decades [14]. Their results showed that the rate of decline in high-income countries was greater than that of middle- and low-income countries (−0.49% versus −0.39% per year) [14].

In the current study, no differences were found between countries for children's mean grip strength. Irrespective of country, grip strength was not related to triceps skinfold thickness; however, grip strength was a weak positive correlate of BMI in Canadian and Mexican children. Because weight gain is associated with increases in both lean body mass and fat mass [35], the positive associations observed were likely driven by a greater lean body mass in the heavier children within Canada and Mexico. We speculate that the positive effects that the increased lean body mass had on muscular fitness in the heavier Canadian and Mexican children were not reflected in higher grip strength values than in Kenyan children because these effects were negated by decreases in physical activity that affected muscle quality (e.g., strength per kg of muscle). It is also possible that insufficient variability in the BMIs of the Kenyan sample resulted in a lack of power to detect meaningful associations.

The low prevalence of overweight and obesity in the Kenyan children (5.6%) examined in this study was expected given their stage of the nutrition and physical activity transitions and previously published data from that country. In particular, the 2003 Kenya Global School-Based Student Health Survey found that only 5.9% of 10–15-year-old boys and girls were overweight or obese [8, 36]. Although Mexico sits at an earlier stage of the nutrition and physical activity transitions than Canada, the prevalence of obesity in the Mexican children (9.2% boys, 8.4% girls) studied here was slightly higher than in the Canadian children (8.6% boys, 7.2% girls). Although this observation is inconsistent with where the two countries currently sit within the nutrition and physical activity transitions, this was not unexpected as these differences are consistent with nationally representative data for the two countries. Specifically, the prevalence of obesity in 5–19-year-old boys and girls in the 2006 Mexican National Health and Nutrition Survey was between 16.5% and 23.3% [37] while the prevalence of obesity in 6–17-year-old boys and girls in the 2004 Canadian Community Health Survey was between 7.5% and 11.1% [38]. The higher rates of obesity in Mexican children may be due to a variety of factors including differences in dietary and physical activity behaviours, biological differences, and how they interact with their environments. Growth stunting (very low height for age) could also be a plausible explanation for the higher obesity rates observed within the Mexican population. However, stunting in Mexico is on the decline. For example, between 1988 and 2006 stunting decreased from 27% to 16% in Mexican children under the age of 5 [39] and results from the 2006 Mexican National Health and Nutrition Survey found that only 9.9% of children between the ages of 5 and 11 were stunted [40]. Furthermore, within the current study no differences were observed between the height of Canadian and Mexican children suggesting that, in the current study, the higher rates of obesity were not likely due to stunting.

Although temporality of relationships cannot be addressed in this study, the relations between the aerobic fitness and body composition measures suggest that low fitness has a greater impact on the body composition of Mexican children than on that of Canadian and Kenyan children. Thus, as Mexico continues to progress through the physical activity transition, wherein their physical activity and fitness levels approach those currently observed in Canada, we can anticipate that the obesity levels in Mexican children will rise at a faster rate than what has occurred in Canada in recent decades. Conversely, as Kenya progresses through the physical activity transition, the increased prevalence of obesity in the population may more closely match what has occurred in Canada. Nonetheless, our findings suggest that it may be inaccurate to project changes in children's body composition in developing countries based on previous trends observed in developed countries. Thus, reproducing preventive physical activity initiatives that have been successful in high-income countries may have varying levels of success in lower-income countries. For example, our findings suggest that more substantial changes in physical activity and fitness would need to occur within Mexican children to have the same body composition benefits observed in predominately non-Hispanic White populations such as Canada. Dietary initiatives may also differ; however, the differential effects of diet on the body composition of children in different countries requires further investigation. 

As with all studies, this one is not void of limitations. Because the Kenya and Mexico testing sites were at schools that did not have a gymnasium, the aerobic fitness testing was performed outdoors where the high temperature and humidity could not be controlled. Altitude also negatively impacts aerobic fitness performance [41], and therefore the VO_2max_ values obtained around the city of Nairobi in the Kenyan children were likely underestimated (though this would only further strengthen our findings). In addition, the aerobic fitness of Canadian children was assessed using the mCAFT test as opposed to the 20 m Shuttle Run Test that was used in Mexico and Kenya. Thus, equations used to estimate VO_2max_ were different for Canadian youth resulting in possible comparability issues. Furthermore, the Mexican and Kenyan samples were convenience samples, which limit the generalizability of the findings, particularly as it pertains to how they may have been influenced by urban/rural status. Approximately 50% of the Kenyan sample was from an urban area, while in the country as a whole only 22% of the population is urbanized [42]. The entire (100%) Mexican sample was from an urban area, while in the country as a whole 78% of the population is urbanized [43]. Even within a country children may sit at different stages of the nutrition and physical activity transitions depending on where they live. In Kenya, for instance, children residing in urban areas are more obese and have lower physical activity and fitness levels than children residing in rural areas [7, 8]. While this urban/rural issue may have influenced the descriptive data, they were unlikely to have influenced the relations between the fitness and body composition measures. That is, when relationships between body composition and fitness measures were assessed by rural and urban dwelling in the Kenyan sample, no significant differences were observed in the intercepts and regression coefficients (data not shown). 

In conclusion, there appear to be differences in the fitness and body composition measures of children from countries that currently sit at different stages of the nutrition and physical activity transitions. While negative relationships between aerobic fitness and obesity were observed in children from all three countries examined in this study, these relationships were more pronounced in Mexican children. This may, in part, explain why the prevalence of obesity was higher in Mexican children than in their Canadian counterparts even though Mexico is at an earlier stage of the nutrition and physical activity transitions.

## Figures and Tables

**Table 1:** Descriptive statistics by sex and country.

	Canada	Mexico	Kenya	Country differences*
Boys				
*N *	374	98	86	
Age, y (95% CI)	10.9 (10.8, 11.0)	11.1 (11.0, 11.3)	11.0 (10.9, 11.2)	None
BMI, kg/m^2^ (95% CI)	19.2 (18.8, 19.6)	19.8 (19.0, 20.5)	16.2 (15.7, 16.7)	M > K, C > K
Underweight (%)	5.9	6.1	44.4	
Normal weight (%)	67.8	54.1	52.8	
Overweight (%)	17.7	30.6	1.9	
Obese (%)	8.6	9.2	0.9	
Height, cm (95% CI)	145.8 (144.8, 146.8)	146.8 (145.2, 148.5)	142.0 (140.4, 143.6)	M > K, C > K
Waist circumference, cm (95% CI)	66.2 (65.1, 67.3)	70.0 (67.8, 72.3)	59.6 (58.5, 60.8)	C < M, M > K, C > K
Triceps skinfold, mm (95% CI)	13.1 (12.5–13.6)	13.3 (12.0–14.5)	7.8 (7.0, 8.6)	M > K, C > K
Grip strength, kg (95% CI)	35.0 (33.9, 36.0)	36.6 (34.7, 38.5)	34.7 (32.0, 37.3)	None
VO_2__max_, mL/kg/min (95% CI)	41.3 (40.1, 42.7)	47.1 (46.1, 48.1)	50.2 (49.0, 51.4)	C < M, M < K, C < K

Girls				
*N *	362	95	93	
Age, y (95% CI)	10.9 (10.8, 11.0)	10.8 (10.7, 11.0)	11.0 (10.8, 11.2)	None
BMI, kg/m^2^ (95% CI)	18.8 (18.4, 19.1)	19.2 (18.3, 20.1)	16.8 (16.2, 17.4)	M > K, C > K
Underweight (%)	6.9	15.8	37.9	
Normal weight (%)	68.2	52.6	53.7	
Overweight (%)	17.7	23.2	5.6	
Obese (%)	7.2	8.4	2.8	
Height, cm (95% CI)	146.1 (145.1, 147.2)	145.6 (143.9, 147.4)	143.6 (142.0, 145.3)	None
Waist circumference, cm (95% CI)	64.7 (63.7, 65.7)	67.1 (64.8, 69.4)	60.4 (58.8, 62.0)	M > K, C > K
Triceps skinfold, mm (95% CI)	13.7 (13.2, 14.2)	13.6 (12.5, 14.7)	10.9 (9.7, 12.1)	M > K, C > K
Grip strength, kg (95% CI)	32.9 (31.9, 33.9)	32.3 (31.6, 34.9)	31.1 (28.7, 33.5)	None
VO_2__max_, mL/kg/min (95% CI)	38.3 (37.1, 39.5)	46.4 (45.5, 47.2)	46.7 (45.7, 47.8)	C < M, C < K

*Country differences identified by nonoverlapping confidence intervals; C: Canada, M: Mexico, and K: Kenya.

**Table 2:** Pearson correlations between the three body composition measures by sex and country (*P* < 0.0001 for all correlations).

	Canada		Mexico		Kenya	
	Waist circumference	Triceps skinfold	Waist circumference	Triceps skinfold	Waist circumference	Triceps skinfold
Boys						
Body mass index	0.95	0.82	0.94	0.84	0.80	0.79
Waist circumference	—	0.79	—	0.83	—	0.62

Girls						
Body mass index	0.94	0.75	0.95	0.88	0.92	0.87
Waist circumference	—	0.71	—	0.87	—	0.82

**Table 3:** Age-adjusted relationship between body composition and aerobic fitness (VO_2 max_) measures by sex and country.

	Boys				Girls			
	Canada	Mexico	Kenya	Differences*	Canada	Mexico	Kenya	Differences*
Body mass index								
*β* (95% CI)	−0.21 (−0.23, −0.19)	−0.43 (−0.57, −0.30)	−0.17 (−0.25, −0.09)	C < M, M > K	−0.22 (−0.27, −0.18)	−0.57 (−0.77, −0.37)	−0.35 (−0.47, −0.22)	C < M, M > K
*R* ^2^	0.51	0.31	0.19		0.47	0.32	0.24	
Predicted BMI at VO_2 max_ 40 mL/kg/min	19.3	22.9	18.0		18.5	22.9	19.3	
Predicted BMI at VO_2 max_ 50 mL/kg/min	17.2	18.5	16.2		16.5	17.2	15.7	
Triceps skinfold								
*β* (95% CI)	−0.32 (−0.39, −0.25)	−0.76 (−0.97, −0.55)	−0.32 (−0.46, −0.18)	C < M, M > K	−0.31 (−0.36, −0.26)	−0.79 (−1.04, −0.55)	−0.75 (−0.97, −0.52)	C < M, C < K
*R* ^2^	0.42	0.31	0.19		0.37	0.33	0.32	
Waist circumference								
*β* (95% CI)	−0.53 (−0.60, −0.48)	−1.29 (−1.66, −0.92)	−0.32 (−0.52, −0.12)	C < M, M > K	−0.63 (−0.76, −0.50)	−1.46 (−1.98, −0.94)	−0.84 (−1.15, −0.53)	C < M
*R* ^2^	0.48	0.34	0.12		0.53	0.32	0.24	

*Country differences identified by nonoverlapping confidence intervals; C: Canada, M: Mexico, and K: Kenya.

**Table 4:** Age-adjusted relationship between body composition and muscular fitness (grip strength) measures by sex and country.

	Boys				Girls			
	Canada	Mexico	Kenya	Differences*	Canada	Mexico	Kenya	Differences*
BMI				None				M > K, C > K
*β* (95% CI)	0.10 (0.00, 0.21)	0.14 (0.06, 0.23)	0.04 (−0.00, 0.08)		0.16 (0.07, 0.24)	0.27 (0.18, 0.37)	0.00 (−0.06, 0.06)	
*R* ^2^	0.10	0.09	0.05		0.15	0.32	−0.02	
Predicted BMI at grip strength of 27 kg	18.5	18.5	15.9		17.8	17.6	16.8	
Predicted BMI at grip strength of 41 kg	19.9	20.4	16.4		20.1	21.5	16.8	
Triceps skinfold				None				M > K
*β* (95% CI)	−0.02 (−0.11, 0.07)	0.13 (−0.02, 0.28)	0.01 (−0.06, 0.08)		0.06 (−0.07, 0.18)	0.27 (0.14, 0.40)	−0.07 (−0.18, 0.04)	
*R* ^2^	0.00	0.02	−0.02		0.01	0.18	0.01	
Waist circumference				None				M > K, C > K
*β* (95% CI)	0.31 (0.09, 0.53)	0.39 (0.15, 0.63)	0.10 (0.00, 0.19)		0.41 (0.18, 0.63)	0.71 (0.45, 0.96)	0.00 (−0.14, 0.15)	
*R* ^2^	0.14	0.09	0.06		0.16	0.32	−0.01	

*Country differences identified by nonoverlapping confidence intervals; C: Canada, M: Mexico, and K: Kenya.
